# Non-compliance is the predominant cause of aspirin resistance in chronic coronary arterial disease patients

**DOI:** 10.1186/1479-5876-6-46

**Published:** 2008-08-29

**Authors:** Kenneth A Schwartz, Dianne E Schwartz, Kimberly Barber, Mathew Reeves, Anthony C De Franco

**Affiliations:** 1 Michigan State University, Department of Medicine, B203 Life Sciences Bldg., East Lansing, MI 48824, USA; 2 Genesys Medical Center, One Genesys Parkway, Grand Blanc, Michigan 48439, USA; 3 Michigan State University, Department of Epidemiology, A220 West Fee Hall, East Lansing, MI 48824, USA; 4 Cardiology Associates, 3219 Clifton Ave., Cincinnati, OH 45220, USA

## Abstract

**Background:**

Our previous publication showed that 9% of patients with a history of myocardial infarction MI. could be labeled as aspirin resistant; all of these patients were aspirin resistant because of non-compliance. This report compares the relative frequency of aspirin resistance between known compliant and non-compliance subjects to demonstrate that non-compliance is the predominant cause of aspirin resistance.

**Methods:**

The difference in the slopes of the platelet prostaglandin agonist (PPA) light aggregation curves off aspirin and 2 hours after observed aspirin ingestion was defined as net aspirin inhibition.

**Results:**

After supposedly refraining from aspirin for 7 days, 46 subjects were judged non-compliant with the protocol. Of the remaining 184 compliant subjects 39 were normals and 145 had a past history of MI. In known compliant subjects there was no difference in net aspirin inhibition between normal and MI subjects. Net aspirin inhibition in known compliant patients was statistically normally distributed. Only 3% of compliant subjects (2 normals and 5 MI) had a net aspirin inhibitory response of less than one standard deviation which could qualify as a conservative designation of aspirin resistance. A maximum of 35% of the 191 post MI subjects could be classified as aspirin resistant and/or non-compliant: 9% aspirin resistant because of non-compliance, 23% non-compliant with the protocol and possibly 3% because of a decreased net aspirin inhibitory response in known compliant patients.

**Conclusion:**

Our data supports the thesis that the predominant cause of aspirin resistance is noncompliance.

## Background

A daily aspirin delays the progression of occlusive atherosclerotic vascular disease [1-5]. Patients with a history of myocardial infarction who are taking aspirin have a 25% decrease in adverse vascular events while patients with increasing symptoms of angina have a 50% decrease in vaso-occlusive events with aspirin therapy [6-8]. However, the rapid progression of symptomatic occlusion in some patients prescribed aspirin has led to the notion that these patients are resistant to the effects of aspirin.

Aspirin resistance has been loosely defined as decreased inhibition of platelets when measured using a platelet function assay or by quantitation of a serum or urinary metabolite of thromboxane B2 and has been described in up 45% of patients [9-12]. The proportion of patients who fit a particular author's definition of aspirin resistance varies according to the method used to assess aspirin's antiplatelet effect as well as the somewhat arbitrary separation of patients into subsets of aspirin resistant and aspirin sensitive patients [13]. Five studies and two meta-analysis show that patients with, "aspirin resistance", have a more rapid progression of their atherosclerotic disease [14-20].

Clinically important causes of aspirin resistance are noncompliance and non-aspirin non-steroidal anti-inflammatory drugs (NANSAIDs) interference with aspirin's effect and other hypothesized mechanisms like increased platelet turnover [21-23]. We have previously reported that approximately 9% of MI subjects who were presumed to be aspirin resistant were aspirin resistant because of noncompliance [24]. Compliance was documented as a key factor in explaining decreased platelet inhibition with aspirin in five other reports (Table 1) [25-29]. In order to properly treat a patient who appears to be aspirin resistant it is important for a clinician to have some estimate as to the likely cause.

**Table 1:** Demonstration of Aspirin non-Compliance by Repeat Testing

		Methods for		
			
N	% non-Compliant	ASA Effect	Repeat Testing for Compliance After	Reference
192	9.0	AA Light Aggregometry	Observed ASA ingestion	[24]
212	14.0	PFA-100	Strict reinforcement of compliance	[27]
203	3.4	Thromboelastography	Hospitalization	[26]
73	16.0	Thromboxane B2: plasma	Admitted to non-compliance	[28]
87	20.0	Collagen Light Aggregometry	Admitted to non-compliance	[29]
678	2.0	AA light Aggregometry	Ex vivo ASA	[25]

We evaluated our aspirin platelet function data to determine what proportion of subjects could be classified as non-compliant and what percentage of subjects could be classified as aspirin resistant from a cause that was independent of compliance [30]. Compliance with both off aspirin and 2 hour post aspirin was confirmed with arachidonic acid (AA) light aggregometry. The protocol required patients to stop aspirin and NANSAIDs for 7 days. Compliance was assured by watching the subjects ingest aspirin and by demonstrating diminished post aspirin AA stimulated platelet aggregation. The degree of aspirin induced inhibition of platelet function was assessed using platelet prostaglandin agonist (PPA) stimulated light aggregations measured when subjects were off aspirin and 2 hours after observed aspirin ingestion [31]. The decrease in aspirin induced platelet response was used to calculate a novel measurement of aspirin effect, net aspirin inhibition. The presented data support the thesis that the predominant cause of aspirin resistance is non-compliance [32].

## Methods

Subjects were contacted by phone, and after a detailed explanation of the study were invited to participate. Prior to the study informed consent was obtained. Inclusion criteria for the MI. patients were hospital admission for a myocardial infarction during the period between 1995 and 2000 and having been prescribed aspirin for at least of one month prior to study. Exclusion criteria were: history of aspirin noncompliance; primary care physician determination that the patient may not be withdrawn from aspirin; history of hemorrhagic cerebral vascular accident; coronary arteritis; thrombocytopenia; known hypercoagulable disorders; use of non-aspirin nonsteroidal anti-inflammatory drugs (NANSAIDs) or cyclooxygenase (COX)-2 inhibitors during the seven days prior to blood draw; uremia/dialysis or a creatinine greater than 3.0 mg/dl; and failure to provide written informed consent. In addition, thirty nine normal subjects who had not taken platelet inhibiting drugs for seven days and had no known vascular or renal disease were studied. Their measurements of platelet response were compared with those obtained from the myocardial infarction patients. Of the 350 subjects who met the study criteria, 250 agreed to participate and 230 completed the study. Of the 230 subjects 45 were excluded from the analysis of compliant subjects because their off aspirin aggregation responses to AA were less than 50% of maximal and one patient who admitted to violating the protocol's stipulation not to take NANSAIDs. These subjects were judged not to be compliant with the protocol's instructions to refrain from ingesting aspirin or NANSAIDs for 7 days. The 184 subjects (39 normals and 145 post myocardial infarction) who were compliant with the protocol were studied as known compliant subjects. The total number of compliant and noncompliant post-MI patients was 191. This study was approved by the Institutional Review Boards at McLaren Medical Center, Flint, MI. and at Ingham Regional Medical Center, Lansing, MI. and was carried out according to the principles of the Declaration of Helsinki.

### Light transmittance aggregometry

Platelet function was measured using light transmittance aggregometry at 2 time points: after stopping aspirin for 7 days (off aspirin); and 2 hours after observed aspirin ingestion (on aspirin). Subjects were instructed to withhold all antiplatelet agents for a period of 7 days. Blood was drawn (off aspirin) for platelet aggregations and the subjects were then instructed to ingest a 325 mg aspirin tablet while the nurse watched. Two hours after the observed ingestion of aspirin (on aspirin) aggregation was assessed for the second time.

Using a 21 g butterfly a minimum of 5 ml of whole blood was drawn by venipuncture into a separate syringe before an additional 9 ml of whole blood was collected using a 10 ml plastic syringe containing 1 ml of 3.2% sodium citrate.

Platelet counts were performed using a Coulter AcT, Miami, Fl. Whole blood was centrifuged 200 × g for 10 minutes for platelet-rich plasma and 2000 × g for 15 minutes for platelet poor plasma.

**Figure 1:**
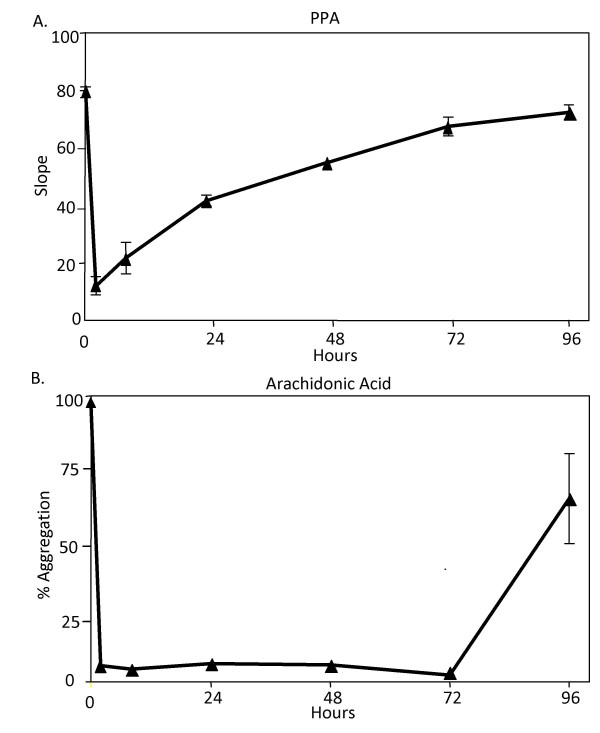
**Note that PPA demonstrates a gradual return of platelet aggregation to normal over 3 days.** This characteristic of PPA stimulated aggregation makes it useful for measuring gradations of aspirin induced platelet inhibition. AA aggregation remains unresponsive for 3 days and returns to normal function between days 3 and 4. AA stimulated platelet aggregations were used to show if aspirin platelet inhibition was present or absent [31].

Light transmittance aggregometry was performed in duplicate using a Helena PACKS4 aggregometer, Beaumont, Tx. The final platelet concentration was adjusted with platelet poor plasma to 150,000/μL. Agreement between the duplicate aggregation curves yielded an intraclass correlation coefficient (ICC) of r = 0.99. (The ICC is a reliability statistic that reflects the extent to which two measurements agree.) To assess the amount of aspirin induced inhibition of platelet function we used the slope of the PPA (30 μM Analytical Control Systems, Fishers, In.) stimulated light aggregometry curve [31]. We defined net aspirin inhibition as the difference between the PPA slopes off and on aspirin. Thus, it was important to know whether the subjects had complied with the protocol's dictum not to take aspirin for 7 days. Light transmittance aggregation with AA (1.0 mM Chronolog, Havertown, Pa.) was used to determine if subjects had refrained from aspirin ingestion for 7 days. AA aggregations were evaluated as percent of maximal aggregation. Normal, non-aspirin exposed, platelets aggregate. While on aspirin platelets exhibit minimal AA aggregation (Figure 1) [31]. In this study subjects with less than 50% of maximal AA stimulated platelet aggregation were judged to be aspirin inhibited. Our prior publication showed that the 50% aggregation delimiter visually separated patients into two groups, normal and aspirin inhibited [24].

### Statistical Analysis

A general linear model was used to evaluate the relationship between the off aspirin PPA slope and the difference between the off and on aspirin PPA slopes. The patient with the largest net aspirin inhibitory response was determined to be an outlier using the Grubbs test for outlying points and was removed from the analysis for normal distribution [33]. The Kolmogorov-Smirnov test was used to determine if the data were normally distributed. Statistical significance was set at a p value of 0.05 or less. All statistical analysis was performed by Alpha Biostats, Reno NV. using SPSS software, Chicago, Il.

The authors had full access to the data and take responsibility for its integrity. All authors have read and agreed to the manuscript as written.

## Results

### Compliant Subjects

The mean age for the 184 known compliant subjects was 63 ± 11 years, with 63% males and 37% females. The mean BMI for myocardial infarction patients was 29.4 with 39 percent having a BMI of more than 31. Thirty nine percent of the coronary artery disease patients had two or more documented myocardial infarctions. The percent of patients with additional risk factors for atherosclerotic vascular disease is presented in Table 2.

**Table 2:** MI Patient Clinical Measures

Mean Age	63 ± 11 years
Mean BMI	29.4 ± 5.6
BMI > 31	39%
Two or More Mis	33%
Smokers	39%
Diabetes	29%
Hypertension (≥ 140/≥ 90)	52%
Total Cholesterol >200	28%
Total Cholesterol >240	6%
HDL < 40	50%
LDL > 130	31%
LDL > 159	9%

NOTES:

Clinical measures were not available for the control subjects.

Not all MI patients have complete clinical measures data.

**Figure 2:**
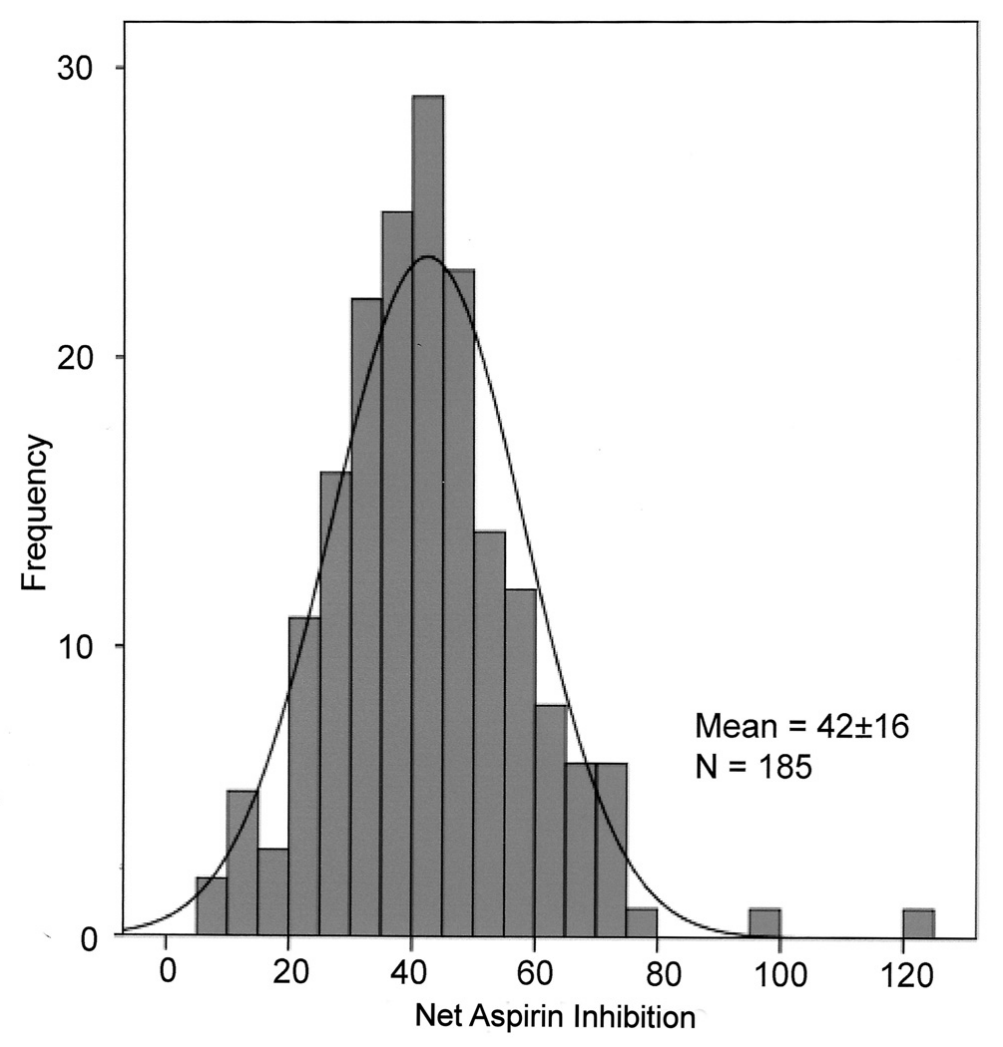
When the single point with the largest aspirin response is removed as a statistical outlier, the net aspirin inhibitory response distribution curve is judged to be normally distributed.

All known compliant subjects had a greater than 50% decrease in aggregation response to AA two hours after observed aspirin ingestion. For these compliant subjects the mean off aspirin PPA aggregation curve slope was 57 ± 14. The mean on aspirin PPA aggregation slope was 15 ± 14. The mean difference between the PPA aggregation slopes off and on aspirin represents net aspirin inhibition and was 42 ± 16. Net aspirin inhibition demonstrated a normal distribution curve (Figure 2). No difference was observed between the 39 normal subjects and the 145 myocardial infarction patients for PPA aggregation slopes off and on aspirin or for net aspirin inhibition (p = 0.61).

**Figure 3:**
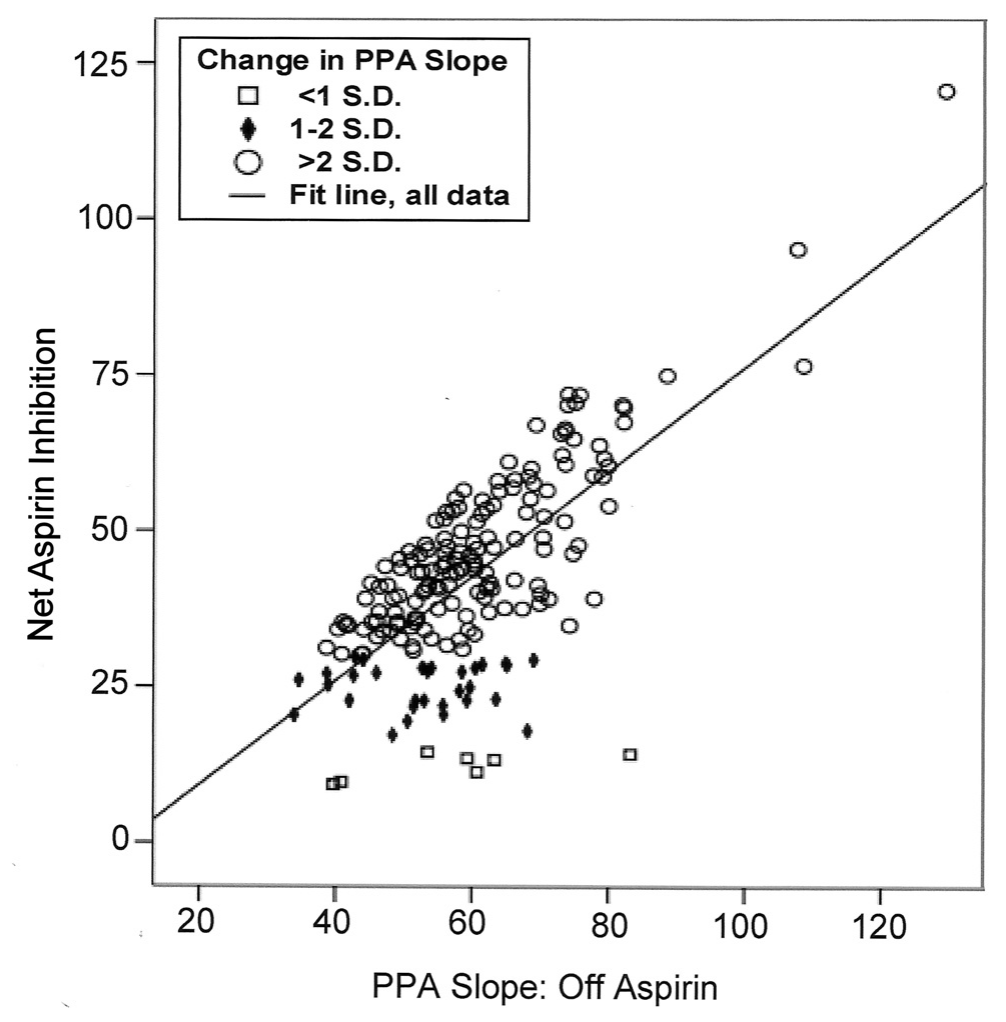
**The seven subjects with less than 1 standard deviation decrease in their on aspirin slopes are depicted by open squares (□), those with a decrease in PPA slope between 1 and 2 standard deviations by solid diamonds (◆) and those with a greater than 3 standard deviation decrease by open circles (○).** A direct relationship is observed between PPA slope off aspirin and the net aspirin inhibitory response. (p < 0.001).

Decreased aspirin response could be defined using the calculated standard deviation. For example, if a decreased aspirin response is defined as the difference in PPA slope between off and on aspirin of one standard deviation or less, 16, then of the 184 compliant subjects 7 (3%) would be classified as having a decreased aspirin response (Figure 3). Of these 7 subjects 5 of 145 (3.4%) had prior myocardial infarctions and 2 of 39 were normal subjects.

The amount of platelet inhibition by aspirin in compliant subjects was related to the off aspirin response (figure 3). As the slope of the off aspirin PPA stimulated aggregation curve increased, net aspirin inhibition increased. Even though net aspirin inhibition was proportional to the off aspirin PPA slope, those subjects with a one standard deviation or less decrease in net aspirin inhibitory response could not be identified by just using their PPA response off aspirin.

### Non-compliant subjects

The mean age for the 191 post MI patients was 61 ± 13 with 66% males and 34% females. As previously reported 16 post-MI patients evaluated after having been prescribed a daily aspirin for at least one month had normal AA light aggregations. However, 2 hours after observed ingestion of 325 mg of aspirin AA aggregations were blocked demonstrating that non-compliance was the cause of the initial non-response to aspirin [24]. An additional 45 patients were judged to be non-compliant with the protocol's stipulation that they stop taking aspirin for seven days because on the 7^th ^day of their proscribed abstinence from aspirin their AA aggregations demonstrated aspirin inhibition. One patient violated the protocol's stipulation not to take NANSAIDs. Of the 191 post-MI patients 62 (32%) were either non-compliant with their prescribed daily aspirin or with the protocols dictum of stopping aspirin and NANSAIDS for 7 days (Table 3).

**Table 3:** Post MI Subjects with Aberrant Platelet Response to Aspirin

	**# of subjects**
**Non-Compliant**	
1. Prescribed daily aspirin	16
2. Protocol directive not to take Aspirin for 7 days	45
3. Protocol directive not to take NANSAIDs for 7 days	1
	
**Protocol Compliant**	
<1 S.D. decrease in Net Aspirin Inhibition	5
Total	67

93% (62 of 67) of aberrant platelet responses to aspirin were due to non-compliance. Among compliant post MI subjects 3% may be classified as aspirin resistant.

## Discussion

After assuring compliance by observing post MI and normal subjects taking their aspirin, we determined that only 7 of 184 (3%) demonstrated decreased platelet inhibition that could be defined as aspirin resistance. There was no difference in net aspirin inhibitory response between normal adults and myocardial infarction patients. This suggests that MI patients who are stable enough to participate in a week-long out patient study have a response to aspirin that is similar to normal subjects.

We used a novel parameter, net aspirin inhibition, to measure the degree of platelet inhibition produced by aspirin. The differences in PPA aggregrometry slopes off and on aspirin demonstrated a normal distribution suggesting that people possibly classified as aspirin resistant in this study were not a distinct population, but represent the lower portion of the bell shaped curve (Figure 2). Because net aspirin inhibition is a continuous variable we thought that designating those subjects with less than a one standard deviation decrease in net aspirin inhibitory response as possibly aspirin resistant seemed reasonable. However, separating aspirin sensitive from aspirin resistant subjects is an arbitrary designation. Perhaps a more clinically useful separation could be derived from a prospective study evaluating net aspirin inhibition as a predictor of future vascular events.

Net aspirin inhibition was related to the off aspirin response (Figure 3). Because aspirin specifically and irreversibly blocks the platelet enzyme cyclooxygenase-1 (COX-1), the variation in observed aspirin inhibition may reflect individual differences in the resting platelet's dependence on activation via the arachidonic acid pathway [31,34,35]. It may be that the subset of subjects with a decreased net aspirin inhibition are those who might benefit from an additional inhibitor of platelet function. This hypothesis needs to be tested in a future study. The information obtained from known compliant subjects confirms that platelet inhibitory response to aspirin is variable, but that a clearly delimited subset of people with a markedly decreased aspirin platelet inhibitory response cannot be defined.

Current clinical considerations for aspirin resistance include patients who are non-compliant, have NANSAID interference with aspirin's ability to inhibit platelets or have a decreased aspirin response. Taken together with our prior report our post MI population of 191 patients had 16 subjects who were noncompliant while taking their daily aspirin another 46 subjects who were non-compliant with the protocol stipulation that they refrain from aspirin ingestion for 7 days and 1 patient who violated the protocols stipulation not to take NANSAIDs [24]. If the above noncompliant subjects are removed from the analysis only 5 known compliant post MI subjects could be identified as having a decreased aspirin effect that could be labeled as aspirin resistant. Of the 67 post MI subjects with an aberrant aspirin effect on platelets 62 (93%) were because of non-compliance with either their prescribed daily aspirin or with the protocol's direction not to take aspirin or an NANSAID for 7 days (Table 2).

Poor platelet inhibition by aspirin is associated with an increase in rate of occlusive atherosclerotic disease [14-18]. A meta-analysis confirmed the increased risk of occlusive vascular disease in patients classified as aspirin resistant [19]. In this meta-analysis the risk for vascular disease was not decreased in those patients who were prescribed Plavix to treat their aspirin resistance. Our data supports the thesis that most of the aspirin resistant patients are resistant because of non-compliance. Perhaps Plavix's lack of benefit in aspirin resistant patients is also because of non-compliance.

Compliance in 1521 myocardial infarction patients was investigated by asking patients to list their medications [36]. Patients who discontinued their daily aspirin one month after their myocardial infarction had a lower survival rate at one year compared to compliant patients 91% vs 97%, p < 0.001 [36]. Strict enforcement of compliance can improve the percentages of patients whose platelets are appropriately inhibited by aspirin [27]. These data suggest that discovering which patient has a problem with compliance could improve the health of patients with CAD.

## Conclusion

Aspirin resistance is an unfortunate descriptor. It suggests an inherited or acquired defect for the ability of aspirin to acetylate platelet COX-1. Our current data interpreted in the context of our prior publication as well as several recent reviews reinforces the importance of patient compliance as a cause of poor inhibition of platelets or aspirin resistance [24,37,32,39]. In view of these findings we suggest that a more accurate nomenclature for patients with poor platelet inhibition by aspirin might rely on the etiology of the poor aspirin response and would accommodate non-compliance, drug interactions and other possible causes.

Platelet stimulation with AA easily stratifies patients as compliant or noncompliant while PPA stimulated aggregometry allows identification of patients whose platelets demonstrate decreased inhibition with aspirin. For the clinician who is confronted with a patient with increasing CAD symptoms the question of aspirin resistance because of compliance or interference with NANSAIDs or decreased aspirin response represents a problem. If the patient is concurrently taking both aspirin and an NANSAID then the appropriate sequence of these two medication needs to be emphasized [21]. Conversely, if the patient truly has a decreased aspirin response, then an additional anti-platelet agent may be warranted. However, our data suggest that the preponderant cause of poor platelet inhibition with aspirin is non-compliance and that the clinician should be encouraged to work to increase the patient's compliance with the prescribed daily aspirin.

## Abbreviations

MI: myocardial infarction; NANSAIDs: non-steroidal anti-inflammatory drugs; AA: light aggregometry; PPA: platelet prostaglandin agonist; COX: cyclooxygenase; CAD: coronary arterial disease.

## Competing interests

The authors declare that they have no competing interests.

## Authors' contributions

KAS designed the study, analyzed the data and wrote the manuscript. DES designed the study, edited the manuscript, evaluated the data and performed platelet laboratory evaluations. KB collected and organized data. MR designed the study and analyzed the data. ACDF designed the study and performed data collection and analysis. All authors read and approved the final manuscript.
